# 

**Published:** 1899-09

**Authors:** 


### CONTENTS.

ORIGINAL COMMUNICATIONS—
THE PATHOLOGY OF FEVER. By J. Clarence Johnson, M.D., Atlanta, Ga.......... 433
MITRAL STENOSIS. By M. F. Carson, M.D., Griffin, Ga.......... 445
AN INTERESTING CASE OF APPENDICITIS. By W. Hal Moncrief, M.D., Atlanta, Ga.......... 452
MEDICAL SUPERSTITIONS. By D. W. B. Conway, M.D., Athens, Ga.......... 456

SOCIETY REPORTS—
ATLANTA SOCIETY OF MEDICINE.......... 464

EDITORIAL NOTES AND COMMENTS—
PROFESSOR LOMBROSO AND VACHER'S BRAIN.......... 479
THE THERAPEUTIC APPLICATIONS OF LIQUID AIR.......... 480
"AMO—I LOVE".......... 481

CONTENTS CONTINUED.......... vi

